# Anisotropic metal growth on phospholipid nanodiscs via lipid bilayer expansion

**DOI:** 10.1038/srep26718

**Published:** 2016-05-24

**Authors:** Jana Oertel, Adrian Keller, Julia Prinz, Benjamin Schreiber, René Hübner, Jochen Kerbusch, Ilko Bald, Karim Fahmy

**Affiliations:** 1 Institute of Resource Ecology, Helmholtz-Zentrum Dresden-Rossendorf, P.O.B. 510119, 01314 Dresden, Germany; 2 Institute of Ion Beam Physics and Materials Research, Helmholtz-Zentrum Dresden-Rossendorf, P.O.B. 510119, 01314 Dresden, Germany; 3 Technical and Macromolecular Chemistry, University of Paderborn, Warburger Str. 100, 33098 Paderborn, Germany; 4 Department of Chemistry, University of Potsdam, Karl-Liebknecht-Strasse 24–25, 14476 Potsdam, Germany; 5 Technische Universität Dresden, Mommsenstraße 13, 01069 Dresden, Germany; 6 BAM Federal Institute of Materials Research and Testing, Richard-Willstätter Strasse 11, 12489 Berlin, Germany

## Abstract

Self-assembling biomolecules provide attractive templates for the preparation of metallic nanostructures. However, the intuitive transfer of the “outer shape” of the assembled macromolecules to the final metallic particle depends on the intermolecular forces among the biomolecules which compete with interactions between template molecules and the metal during metallization. The shape of the bio-template may thus be more dynamic than generally assumed. Here, we have studied the metallization of phospholipid nanodiscs which are discoidal particles of ~10 nm diameter containing a lipid bilayer ~5 nm thick. Using negatively charged lipids, electrostatic adsorption of amine-coated Au nanoparticles was achieved and followed by electroless gold deposition. Whereas Au nanoparticle adsorption preserves the shape of the bio-template, metallization proceeds via invasion of Au into the hydrophobic core of the nanodisc. Thereby, the lipidic phase induces a lateral growth that increases the diameter but not the original thickness of the template. Infrared spectroscopy reveals lipid expansion and suggests the existence of internal gaps in the metallized nanodiscs, which is confirmed by surface-enhanced Raman scattering from the encapsulated lipids. Interference of metallic growth with non-covalent interactions can thus become itself a shape-determining factor in the metallization of particularly soft and structurally anisotropic biomaterials.

In the last two decades, the metallization of biomolecules has received considerable attention as the resulting biomolecule-templated metal nanostructures have great promise for applications in nanoelectronics and plasmonics. A variety of biomolecular complexes including lipid tubules^1^, microtubules^2^, amyloid fibrils^3^, S-layers^4^, and especially DNA^5^,^6^,^7^,^8^,^9^,^10^,^11^ have been used as templates for metallization. Consequently, a number of techniques have been developed for the deposition of different metals on the biomolecular templates including silver^3^,^6^, gold^3^,^8^,^10^, copper^9^, nickel^1^,^2^, cobalt^1^,^2^, platinum^7^, and palladium^4^,^5^,^11^. With few exceptions^3^, most of these methods rely on the activation of the biomolecules by a specific immobilization of metal ions, followed by a reduction step which leads to the formation of metallic nanoclusters along the template. These clusters can then be fused together by electroless deposition so that a continuous metallic nanostructure is obtained.

Recently, Schreiber *et al*. reported a different approach that does not rely on a direct binding of metal ions but rather makes use of the negative charge of the DNA backbone to assemble metallic nanoclusters along the DNA strands^12^. To this end, the authors incubated differently shaped DNA nanostructures with positively charged gold nanoparticles (AuNPs) which were then, due to electrostatic interactions with the phosphate groups in the DNA backbone, immobilized on the DNA surface. By electroless gold deposition, the AuNPs could again be fused together, so that DNA-templated AuNPs of defined shape were formed. In this work, we have adapted this approach^12^ and applied it to another biomolecular nanostructure of considerable importance, namely phospholipid nanodiscs (NDs).

Phospholipid NDs are water-soluble planar phospholipid bilayer particles surrounded by two copies of an amphipathic helical protein (membrane scaffold protein, MSP) derived from apolipoprotein ApoA1^13^. NDs exhibit a very well-defined size between 10 and 20 nm depending on the MSP variant used. This allows for the incorporation of single membrane proteins into the NDs^14^. Despite the confinement of the phospholipid bilayer by the MSP, NDs provide a native-like environment for membrane proteins which maintain their native structure and function^15^,^16^. Therefore, phospholipid NDs represent promising systems for the structural investigation of membrane proteins and their interaction with drug molecules by a variety of spectroscopic techniques including Raman scattering^17^ and surface-enhanced infrared spectroscopy (SEIRS)^18^. Furthermore, NDs can also be used as building blocks for the self-assembly of larger hierarchical nanostructures. For instance, DNA-modified NDs have been synthesized by insertion of cholesterol-modified DNA oligonucleotides into the membrane which assembled upon DNA hybridization into wire-like bionanostacks^19^. By utilizing the polyhistidine tag of the MSPs, these bionanostacks could then be used as scaffolds for the controlled arrangement of gold nanoparticles^20^.

Here, we investigate the metallization of NDs and elucidate the fate of the bio-template during metallization by spectroscopic and microscopic techniques. In contrast to DNA, NDs are naturally soft and more fragile due to the confining protein belt, and thus may get deformed and even disrupted during metallization. Therefore, studying the internal structure of the NDs during metallization will be indispensable for understanding the metallization process and potentially exploiting unique features of deformable matrices for the template-directed 2D or 3D growth of metallic layers and especially metal-insulator stacks.

We have followed the metal growth on NDs *in situ* by atomic force, scanning electron, and transmission electron microscopy, as well as optical spectroscopy. Infrared spectroscopy has been employed to detect metallization-induced structural changes of the constituents of the NDs. These investigations reveal that metallization proceeds in the form of a template-directed lateral growth of the AuNPs immobilized on the lipid headgroups which leads to a significant lateral expansion of the ND core. Using infrared spectroscopy and surface-enhanced Raman scattering (SERS) measurements of the AuNDs, we assess their inner structure and demonstrate strong Raman enhancement of the enclosed lipidic phase.

## Results and Discussion

**Figure 1:**
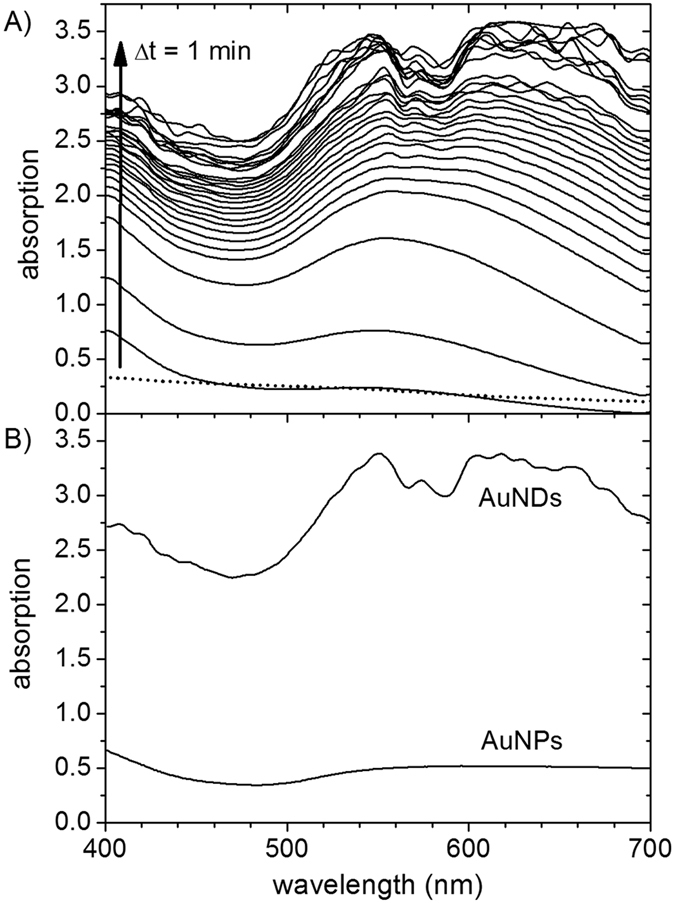
(**A**) UV-Vis spectra of AuNDs during electroless deposition recorded at time increments Δt = 1 min. The dotted line represents the spectrum of the AuNP-decorated nanodiscs before electroless deposition. (**B**) UV-Vis spectra of AuNDs and 1.4 nm AuNPs after 21 min of electroless deposition.

Negatively charged NDs have first been assembled from the MSP variant MSP1D1^21^ and negatively charged DMPG (1,2-dimyristoyl-*sn*-glycero-3-phospho-1′-rac-glycerol) lipids. The negatively charged DMPG-lipid NDs were then mixed with positively charged amine-coated AuNPs with a diameter of 1.4 nm. Due to the attractive electrostatic interactions with the DMPG head groups, AuNPs got immobilized on both sides of the confined lipid bilayer. After removal of unbound AuNPs by spin filtering, electroless gold deposition was performed and followed *in situ* using UV-Vis spectroscopy. Figure 1A shows the obtained absorption spectra recorded at time increments of Δt = 1 min. The absorption is increasing drastically with deposition time at all wavelengths investigated, indicating the continuous deposition of metallic gold. However, the increase of absorption is considerably slowed down already after a few minutes until almost no further increase is observed for t > 20 min which indicates the depletion of gold ions in the solution. Already after 1 min of deposition, a strong increase of absorption at wavelengths below 440 nm is observed, corresponding to the interband absorption of gold^22^. After 2 min, a second rather broad absorption peak appears around 550 nm, *i.e.*, in the spectral region of the typical plasmon resonances of AuNPs. At longer deposition times, the 550 nm peak becomes narrower and the absorption at wavelengths above 600 nm strongly increases which probably indicates the formation of larger aggregates of AuNDs. The absorption bands measured over the first 10 min scale with the amount of the respective materials and show the successive increase of fully metallized AuNDs in relation to the initially formed metallic gold clusters. At later times, the instrument response saturates, which leads to increased noise and prevents quantitating the formation of aggregates.

Figure 1B compares UV-Vis absorption spectra of AuNDs and pure AuNPs without NDs both after 21 min of electroless deposition. The pure AuNPs show a much lower total absorption and do not exhibit a pronounced plasmon absorption peak. Although the initial amount of gold in solution was identical for the two samples, the colloidal AuNPs have a very small size of 1.4 nm and only a rather small amount of gold was deposited. Since the extinction coefficient of AuNPs in this size range increases about one order of magnitude when the diameter of the particles is doubled^23^, the larger AuNDs also have a much larger extinction. The appearance of the plasmon resonance peaks in the AuND sample is therefore also accompanied by a change of color: the initially clear solution turns purple during deposition when the individual AuNPs are fused together.

**Figure 2:**
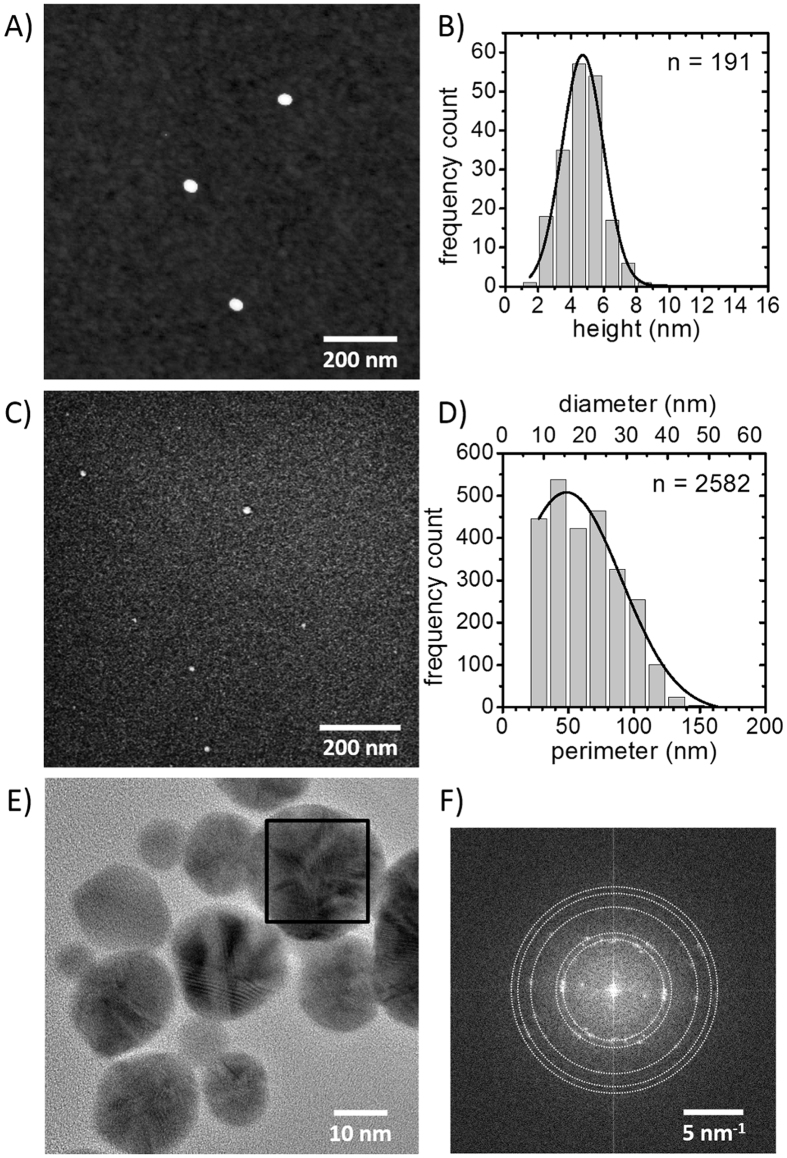
Structural characterization of the AuNDs. (**A**) AFM image of AuNDs (height scale 6 nm) and (**B**) corresponding histogram of the AuND height. The solid line in (**B**) corresponds to a Gaussian fit yielding a mean AuND height of 4.7 nm and a FWHM of 2.5 nm. A total of n = 191 AuNDs have been analyzed. (**C**) SEM image of AuNDs and (**D**) corresponding histogram of the AuND perimeter/diameter. The solid line in (**D**) corresponds to a Gaussian fit yielding a mean AuND diameter of 15.5 nm and a FWHM of 26.5 nm. A total of n = 2582 AuNDs have been analyzed. (**E**) HR-TEM image of AuNDs. (**F**) Fast Fourier Transform (FFT) of the square region indicated in (**D**). As a guide to the eye, the Au diffraction rings are marked by dotted circles.

The geometric and crystalline structure of the AuNDs has been assessed by atomic force and electron microscopy. Figure 2A shows an atomic force microscopy (AFM) image of AuNDs immobilized on a silicon surface. Three AuNDs with almost circular shape and similar diameters are clearly observed. Due to the convolution with the AFM tip, however, only the heights of the AuNDs have been determined from the AFM images. The histogram of heights given in Fig. 2B reveals a rather narrow distribution. The Gaussian fit of the distribution indicated by the solid line in Fig. 2B is centered at a height of 4.7 nm and has a full width at half maximum (FWHM) of 2.5 nm. This height value is very similar to the height of non-metallized DMPG-lipid NDs of 5.0 nm as determined by AFM (see Supplementary Information for details) and agrees fairly well with the reported thickness of DMPG bilayers in solution^24^.

In order to accurately characterize the diameter of the AuNDs, scanning electron microscopy (SEM) images of the same sample have been taken. In the SEM image shown in Fig. 2C, AuNDs are clearly resolved due to the high Z contrast between gold and silicon. A stack of 400 individual SEM images has been analyzed to determine the lateral size of the AuNDs. To this end, a lower threshold of 50 nm^2^ has been introduced corresponding to a lower AuND diameter of 8 nm, in order to exclude residual free AuNPs from the analysis. The perimeter/diameter histogram and the Gaussian fit shown in Fig. 2D reveal a rather broad distribution centered at a mean AuND diameter of 15.5 nm with a FWHM of 26.5 nm. The fact that AuNDs with diameters larger than 40 nm are observed suggests the presence of aggregates of AuNDs, as already observed in the UV-Vis spectra. Aggregates of a few AuNDs may form during deposition and incubation and get immobilized on the silicon surface. On the other hand, the decoration of the negatively charged DMPG-lipid NDs with the positively charged AuNPs may lead to a local charge inversion on the surface of some of the NDs. This charge inversion might then facilitate the AuNP-mediated aggregation of the NDs prior to electroless deposition.

NDs assembled from MSP1D1 exhibit a mean diameter of 9.7 nm as determined by small angle X-ray scattering^21^. Therefore, the rather dominant peak of the size distribution in Fig. 2D at a diameter of 15.5 nm most likely corresponds to the diameter of the AuND monomer. This increase in diameter by ~6 nm is particularly noteworthy as no increase in ND thickness is observed by AFM (Fig. 2B).

The crystalline structure of the AuNDs has been assessed by high-resolution transmission electron microscopy (HR-TEM). The HR-TEM image in Fig. 2E shows AuNDs of different size. The microstructure of the AuNDs can be clearly resolved, revealing their polycrystalline nature. This is even more evident from the Fast Fourier Transform (FFT) given in Fig. 2F, which has been calculated from the square region indicated in the TEM image, *i.e.*, from a single AuND. The FFT consists of several spots arranged on concentric circles evidencing the existence of several crystallites in a single AuND. In particular, the observed spots correspond to lattice spacings of 2.35 Å, 2.04 Å, 1.45 Å, 1.24 Å, and 1.16 Å, which can be assigned to the (111), (200), (220), (311), and (222) lattice planes of gold, respectively. Interference of lattice fringes from two crystallites can lead to Moiré fringes, explaining the two inner peaks in the FFT corresponding to a spacing of 3.79 Å. Although the growth of the AuNPs due to electroless gold deposition proceeds epitaxially^25^, the fact that the AuNPs are immobilized with random orientation on the ND leads to the formation of grains and thus polycrystalline AuNDs.

**Figure 3:**
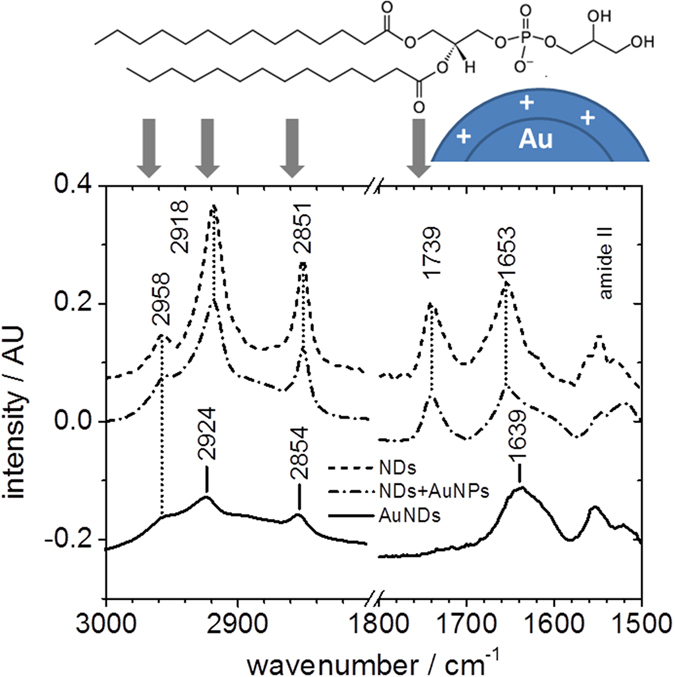
ATR-FTIR spectra of NDs at different stages of the metallization process: pure non-metallized NDs (broken line), NDs with immobilized AuNPs before gold deposition (dash-dotted line), fully metallized AuNDs (solid line). All spectra are corrected for background absorption of the respective buffer solutions. The amide II absorption range is shown for completeness but variability in buffer absorption in this range prevents a more precise frequency assignment. The chemical structure of DMPG is shown on top of the spectra. Arrows indicate the IR-spectral range in which the structural elements show major absorption bands. The putative local interaction with positively charged gold particles before electroless metal deposition is symbolized by the blue sphere.

The state of the organic material within the AuNDs was assessed by attenuated total reflection Fourier transform infrared (ATR-FTIR) spectroscopy. As can be seen in Fig. 3, the association of AuNPs with the DMPG-reconstituted NDs does not cause significant changes in the infrared absorption spectrum. The lipid ester and the amide I absorption peaks at 1739 cm^−1^ and 1653 cm^−1^, respectively, are visible in both preparations. Likewise, the symmetric (2851 cm^−1^) and antisymmetric (2918 cm^−1^) CH_2_ stretching modes are not affected by the electrostatic association of the NDs with the AuNPs. In contrast, extensive spectral changes are observed after electroless gold deposition. The lipid ester carbonyl stretching mode has almost completely disappeared which is indicative of the quenching of its transition dipole moment. Polarization-dependent selection rules of vibrational modes near metallic surfaces^26^ can lead to strong alterations of the relative intensities of absorption bands as is known from reflection absorption infrared spectroscopy of lipids on gold support^27^. Only those vibrational modes are observed that have a transition dipole moment perpendicular to the metal surface. Also the CH_2_ stretching modes are affected by the metallization. The peak intensity is again reduced and an increased frequency of the symmetric and antisymmetric stretching modes observed. Such a frequency up-shift is typical of decreased packing interactions between acyl chains^28^. Remarkably, the frequency of the antisymmetric CH_3_ stretching mode of the acyl chain termini at 2958 cm^−1^ is affected neither by electrostatic adsorption of Au nanoparticles nor by the electroless Au depositions. This suggests that the acyl chain ends in the center of the bilayer do not form inter-leafed structures or become exposed to aqueous or metallic phases during the metallization process. Finally, the broadening and the shift of the amide I mode from 1653 to 1639 cm^−1^ evidences the unfolding of the helical secondary structure of the MSP. The ensemble of the spectral changes suggests an invasion of metallic gold into the sub-headgroup region of the lipids, thereby pushing acyl chains apart. As a consequence of this lateral expansion, the MSP gets stretched longitudinally, leading to the unfolding of its helical structure. In this picture, gold grows predominantly in a lateral manner between lipid headgroups but also towards the center of the bilayer, such that the acyl methylene absorption becomes partially suppressed. Remarkably, the growth proceeds without increasing the thickness of the NDs as observed by AFM (Fig. 2A,B). This demonstrates that the original bilayer thickness, rather than its circumference, is the predominant template dimension that governs metal growth and which becomes inherited by the final metallic particles. The evidenced preservation of organic material in the AuNDs, however, raises the question to which extent non-metallic voids or gaps persist in the AuNDs.

**Figure 4:**
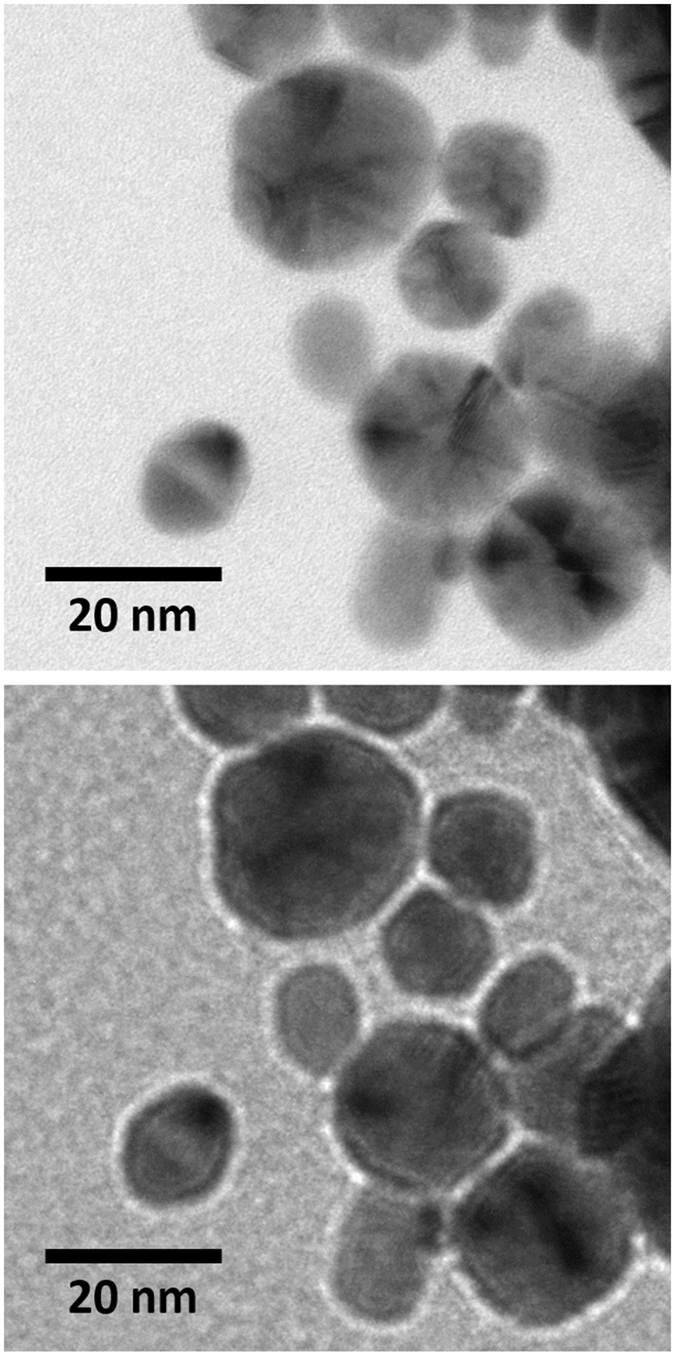
Bright-field TEM images of AuNDs taken in focus (upper image) and with an underfocus of 1.5 μm (lower image).

In an attempt to verify the presence of internal gaps in the AuNDs as suggested by the ATR-FTIR spectra in Fig. 3, the so-called Fresnel contrast has been utilized in TEM imaging^29^. In this imaging mode, Fresnel fringes can be generated wherever the inner potential of the sample changes abruptly by imaging this particular region out of focus. Hence, this technique is particularly useful for imaging voids and embedded bubbles. Figure 4 shows TEM images of the same sample region imaged in focus and with an underfocus of 1.5 μm. In the latter image, Fresnel fringes are clearly visible surrounding the shapes of the AuNDs. Directly inside the AuNDs, however, no clear fringes are visible. There are thus no distinct hints for pores inside the AuNDs. However, disc-shaped internal gaps within sandwich-like Au structures do not provide a strong change in the inner potential and can thus not be resolved by this technique. Therefore, Fresnel imaging in combination with the FTIR results suggests that the AuNDs consist of two parallel gold discs that have partially grown into the membrane region and are separated by a small gap centered at the interface of the two lipid leaflets.

**Figure 5:**
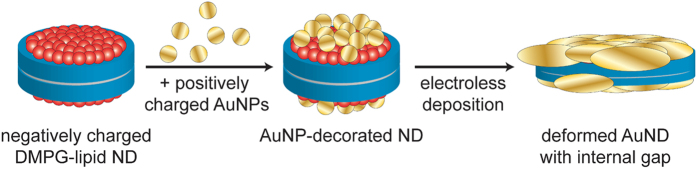
Pathway of ND metallization resulting in deformed AuNDs with internal gaps.

The pathway of ND metallization as deduced from above experimental data is summarized in Fig. 5. Positively charged AuNPs of 1.4 nm diameter are immobilized on both surfaces of the confined DMPG-lipid bilayer due to electrostatic interactions with the negatively charged lipid headgroups. This immobilization affects neither the MSP structure nor the state of the lipids within the NDs as observed in the ATR-FTIR spectra in Fig. 3. During electroless deposition, the immobilized AuNPs grow in size and fuse together. However, instead of isotropic AuNP growth in all directions, the microscopic and spectroscopic data suggest a template-directed lateral growth which leads to a significant increase in ND diameter from about 9.7 to 15.5 nm while the thickness remains largely unaffected (see Fig. 2A–D). Since the observed shift in the amide I band to lower wavenumbers evidences the denaturation of the MSP due to metallization, it appears likely that this lateral growth of the AuNPs leads to a deformation of the ND core of the AuND as depicted in Fig. 5. Although the ATR-FTIR spectra further show the growth of the AuNPs into the membrane region, growth stops before a fully metallized AuND is formed, leaving the AuNDs with an internal, probably lipid-containing, gap. The lack of clear Fresnel fringes inside the AuNDs in the TEM image of Fig. 4 further suggests that this internal gap is not fully embedded in the grown gold matrix but rather represents a thin spacer between two approximately parallel gold discs of about 2 nm thickness.

Similar nanoscale gaps have been observed after electroless deposition onto DNA coated AuNPs^30^. The resulting Au@Au core@shell nanoparticles exhibiting internal gaps of about 1 nm thickness were found to be SERS active due to a strong electromagnetic field enhancement in the nanogaps upon laser irradiation. Therefore, we performed Raman measurements on native NDs and metallized AuNDs in order to evaluate whether the AuNDs are SERS active.

**Figure 6:**
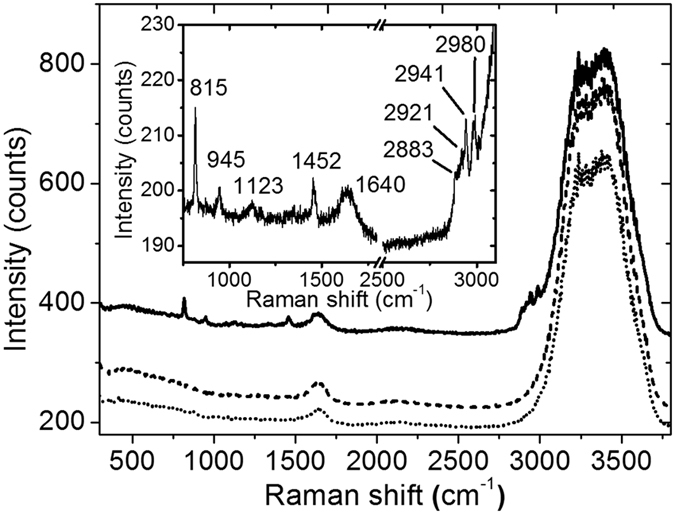
Raman spectra of AuNDs (~320 nM, solid line), non-metallized DMPG-lipid NDs (400 nM, broken line), and pure buffer (dotted line) obtained under the same conditions. The inset shows the relevant parts of the AuND Raman spectrum at high resolution with the wavenumbers of the individual Raman peaks indicated. The excitation wavelength was 532 nm.

The obtained Raman spectrum of the non-metallized NDs (broken line in Fig. 6) consists of some very intense peaks in the range from about 2900 cm^−1^ to about 3700 cm^−1^ and a small peak at 1640 cm^−1^, which are identified as OH stretching and bending modes of water and TRIS in the buffer. An almost identical spectrum is obtained for pure buffer without any NDs (dotted line in Fig. 6). In the case of the metallized AuNDs (solid line in Fig. 6), however, several additional peaks are observed in the spectral regions from 800 cm^−1^ to 1500 cm^−1^ and from 2800 cm^−1^ to 3000 cm^−1^ which are characteristic for phospholipids^31^. The bands at 2800 cm^−1^ to 3000 cm^−1^ correspond to the CH stretching modes of the methylene groups, and the pronounced band at 1452 cm^−1^ is assigned to the CH_2_ scissoring mode^31^. The signal around 1123 cm^−1^ consists most likely of different contributions and is attributed to C-C and PO_2_^−^ stretching modes. The bands at 815 and 945 cm^−1^ originate most likely from the MSP. The band at 945 cm^−1^ can be attributed to the N-C_α_-C mode while the band at 815 cm^−1^ might originate from tyrosine^32^. Since these modes are not observed in non-metallized NDs and taking further into account that the AuNDs were slightly less concentrated, the appearance of these peaks results from a Raman enhancement in the gap region of the metallized DMPG-lipid NDs. This Raman enhancement thus is further evidence for the presence of internal gaps in the AuNDs.

## Conclusion

In the present work, DMPG-lipid NDs have been metallized using electroless gold deposition with colloidal AuNPs as seeds. The seeding process was enabled by the electrostatic interaction of the positively charged AuNPs with the negatively charged DMPG headgroups. UV-Vis spectroscopy revealed the rapid growth of the AuNDs within the first few minutes of deposition, while growth arrest was observed after about 20 min due to the depletion of ionic gold in the solution. The ND efficiently restricts metallization to two-dimensional growth by a mechanism that involves the expansion of the hydrophobic core of the particle at essentially constant thickness. Lateral deformability appears to be the crucial factor for the anisotropic metallization. The obtained discoidal AuNDs are polycrystalline and have a mean height and diameter of 4.7 nm and 15.5 nm, respectively, as determined by atomic force and electron microscopy. The AuNDs furthermore exhibit significant Raman enhancement of the enclosed lipidic phase and thus represent attractive SERS-active substrates.

## Methods

### Assembly of negatively charged DMPG-lipid nanodiscs

The protocol for the phospholipid nanodisc assembly was adapted from the original publication^13^. In order to enable the metallization of the nanodiscs, however, negatively charged 1,2-dimyristoyl-*sn*-glycero-3-phospho-(1′-rac-glycerol) (DMPG; Avanti Lipids) was used. A completely dried DMPG lipid film was solubilized in buffer A, containing cholate as detergent twice the concentration of the lipid, and sonicated until a clear solution was obtained. The respective lipid/sodium cholate solution and MSP1D1 (see Supplementary Information) were mixed to yield a final concentration of 12 mM lipid and 0.2 mM MSP1D1. The mixture was incubated for 1 h at 25 °C. The detergent was removed by Detergent Removal spin columns (Pierce). The size and the homogeneity of the DMPG-lipid NDs were verified by size exclusion chromatography (see Figure S1).

### Metallization of DMPG-lipid nanodiscs

The metallization process of phospholipid nanodiscs was adapted from the protocol of Schreiber *et al*. who applied it to DNA origami nanostructures^12^. For the whole metallization procedure, buffer A was used in a 1:10 dilution (5 mM Tris–HCl, 20 mM NaCl, pH 7.4). Negatively charged DMPG-lipid nanodiscs (400 nM) were incubated with positively charged, amine-coated 1.4 nm nanogold particles (Nanoprobes) at a final concentration of 1.5 μM for one hour at room temperature. After incubation, the sample was spin filtered twice through an Amicon Ultra−0.5 mL filter (100 kDa MWCO, Millipore) to remove excess nanogold and diluted 1:5 in buffer A to get a final concentration of approximately 100 nM nanodiscs decorated with Au-particles. The controlled growth of the nanogold particles was achieved by electroless gold deposition from solution using the gold enhancement kit (GoldEnhance LM, Nanoprobes) following the instructions of the supplier and mixed 1:1 with the Au-seeded nanodisc sample.

### UV-Vis spectroscopy

The metallization process was monitored over time with a Lambda 35 UV-Vis spectrometer (Perkin Elmer) in the wavelength range between 400 and 700 nm using quartz cuvettes with a path length of 1 cm. The absorption spectra from Au-seeded nanodiscs without gold enhancement and the enhancement mixture without nanodiscs were recorded as controls and buffer A as reference.

### Atomic force microscopy

The successful assembly of DMPG-lipid nanodiscs has been verified by atomic force microscopy (AFM). For immobilization, the DMPG-lipid nanodisc sample was diluted 1:10 in buffer A, then mixed 1:25 with 10 mM MgCl_2_ solution and incubated for 3 min on a freshly cleaved mica substrate. After incubation, the sample was rinsed with Milli-Q water and dried in a stream of nitrogen. For the AFM and scanning electron microscopy (SEM) characterization of the metallized nanodiscs, silicon wafers were used as substrates. Directly before immobilization, the substrates were cleaned for 3 min in an oxygen plasma, rinsed with ethanol and Milli-Q water, and dried in a stream of nitrogen. The metallized nanodisc sample was mixed 1:1 with 10 mM MgCl_2_ and incubated on the silicon wafer for at least one hour in a humidity chamber. The wafer was then carefully rinsed with Milli-Q water and dried in a stream of nitrogen. AFM imaging was performed using a Bruker Multimode8 scanning probe microscope operated in tapping mode in air. Tap150Al-G soft tapping cantilevers (Budget Sensors) were used with a nominal force constant of 5 N/m, a resonance frequency of 150 kHz, and a tip radius <10 nm. The AFM images were analyzed using Gwyddion image processing software^33^.

### Scanning electron microscopy

Scanning electron microscopy (SEM) was performed using a RAITH 150^TWO^ electron beam writer. A total of 400 images was recorded using the Inlens-SE detector. The images were arranged in a 20 × 20 matrix with 2 μm center-to-center distance between each image. A magnification of 100 000 and a beam energy of 10 keV was used. To determine the diameters of the individual nanodiscs, an automated particle analysis was performed with WCIF-ImageJ^34^. To this end, the images were converted into binary images by introducing a grayscale threshold of 130. Then the perimeters of the particles with areas between 50 and 1300 nm^2^ were determined and converted into diameters assuming circular shapes.

### Transmission electron microscopy

Samples for transmission electron microscopy (TEM) were prepared by incubating a drop of metallized nanodiscs on a carbon-coated (15 nm thickness) copper grid for several minutes. After incubation, excess liquid was blotted away with a tissue and the grid was air dried. TEM images were obtained using an image-corrected FEI Titan 80–300 microscope operated at 300 kV accelerating voltage.

### Fourier transform infrared spectroscopy

Spectra were recorded with an IFS 66v spectrometer (Bruker) equipped with a liquid nitrogen-cooled MCT detector. Samples (10 μL of pure NDs, AuNP-decorated NDs, and metallized AuNDs) were dried on a diamond attenuated total reflectance (ATR) cell (Resultec). The 1:10 diluted buffer A (5 mM Tris–HCl, 20 mM NaCl, pH 7.4) was used as reference for the pure and the AuNP-decorated NDs while as the AuND reference, a sample of AuNPs incubated with gold enhancement solution was used. 256 interferograms were recorded at a resolution of 2 cm^−1^ and averaged to obtain absorption spectra in reference to the pure ATR crystal.

### Raman spectroscopy

For Raman measurements a drop of the corresponding was applied to a freshly cleaned glass slide. Raman measurements have been performed using a confocal Raman microscope (WITec 300α) equipped with an upright optical microscope. The excitation laser light at 532 nm was coupled into a single-mode optical fiber and focused through a 100x objective (Olympus MPlanFL N, NA = 0.9) to a diffraction-limited spot of about 1.3 μm^2^. The laser power was set to 13 mW and the integration time was 10 s for all measurements. Each spectrum has been obtained by an average of three accumulations. The spectra that cover a broad range of wavenumbers have been recorded using a grating with 600 g/mm whereas for the more detailed measurement a grating with 1800 g/mm has been used.

## Additional Information

**How to cite this article**: Oertel, J. *et al*. Anisotropic metal growth on phospholipid nanodiscs via lipid bilayer expansion. *Sci. Rep.* **6**, 26718; doi: 10.1038/srep26718 (2016).

## Supplementary Material

Supplementary Information
