# Supplementary material for: Anisotropic metal growth on phospholipid nanodiscs via lipid bilayer expansion
Source: Sci Rep. 2016 May 24;6:26718. doi: 10.1038/srep26718 (PMC4877634; doi:10.1038/srep26718)
Supplement: Supplementary Information [file srep26718-s1.pdf]

Supplementary Information

Anisotropic metal growth on phospholipid nanodiscs via lipid bilayer expansion

Jana Oertel, Adrian Keller, Julia Prinz, Benjamin Schreiber, René Hübner, Jochen Kerbusch, Ilko Bald and Karim Fahmy

Expression and purification of MSP1D1

The standard expression of MSP1D1[1] was adapted from literature.[2] Briefly, the MSP1D1-pET28a plasmids were grown over night in BL21Gold (DE3) cells (Agilent Technologies) at 37°C in double strength YT medium containing 50 µg/ml kanamycin. After induction with 0.3 mM isopropyl-β-D-thiogalactopyranoside, the temperature was decreased to 28°C. The cells were harvested 4 h later, frozen in liquid nitrogen, and stored at -80°C until further use. For purification, cells were resuspended in buffer A (50 mM Tris–HCl, 200 mM NaCl, pH 7.4) containing protease inhibitors (Roche Applied Science) and lysed twice with a French Press. Cell debris was removed by centrifugation (10 000 g, 50 min, 4°C). Imidazole was added to the supernatant to a final concentration of 25 mM. The sample was loaded onto a Ni-NTA column (GE-Healthcare), equilibrated with buffer B (buffer A containing 25 mM imidazole). The column was washed with buffer B, and the protein was eluted with 5 ml buffer C (buffer A containing 250 mM imidazole), followed by 5 ml buffer D (buffer A containing 500 mM imidazole). Fractions containing MSP1D1 were identified by SDS-PAGE. Imidazole was removed by a desalting step (PD10 column, equilibrated with buffer A; GE Healthcare) and concentrated using Vivaspin4 columns (Satorius). The final protein concentration was determined by measuring the absorbance at 280 nm with a NanoDrop spectrophotometer using a calculated extinction coefficient of 21 430 M^{-1} cm^{-1} and a calculated molecular weight of 24.793 kDa (ProtParam, ExPASy). Purified MSP1D1 was frozen in liquid nitrogen and stored at -80°C until further use.

Characterization of non-metallized DMPG-lipid nanodiscs

The size and the homogeneity of the DMPG-lipid NDs were verified by size exclusion chromatography (Figure S1) and AFM (Figure S2). The mean height of the non-metallized DMPG-lipid nanodiscs was determined from the height distribution function of the AFM image which exhibits two peaks (see Figure S2, right). The peak at a height of about 1.6 nm corresponds to the bare mica surface while the one at about 6.6 nm corresponds to the nanodiscs. The difference of these peaks thus yields the mean height of the non-metallized nanodiscs, *i.e.*, 5.0 nm, which is very similar to the reported thickness of DMPG bilayers in solution.[3]

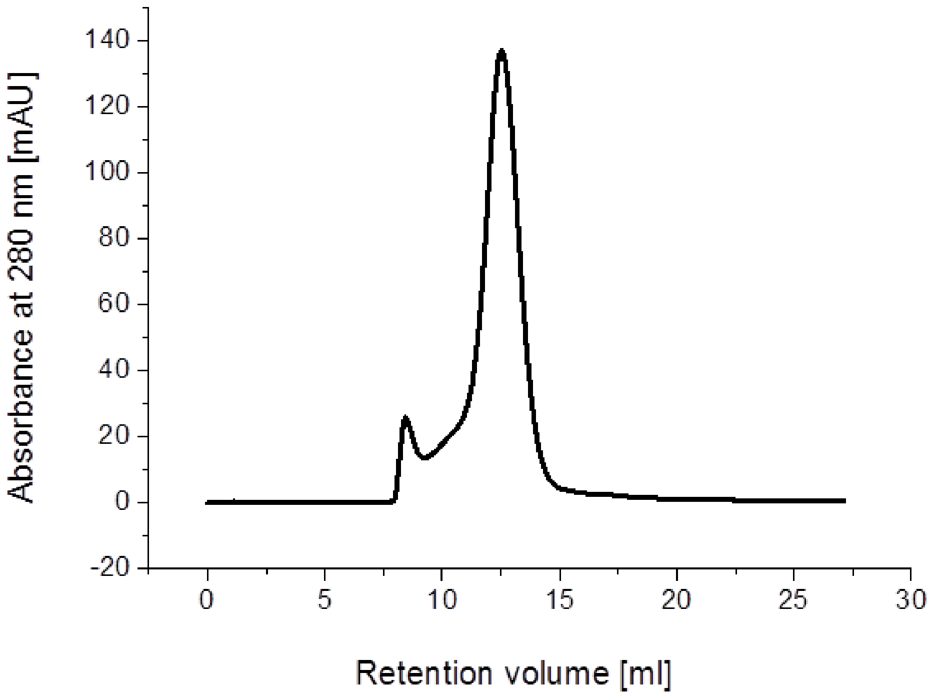

Figure S1: *Size exclusion chromatogram of DMPG-lipid NDs, monitoring the protein absorbance of MSP1 at 280 nm. NDs eluted at V = 11.5 – 13.5 ml, while lipid-protein aggregates eluted at V = 8.3 – 9.1 ml. A Superdex 200 10/300 GL column from GE Healthcare was used.*

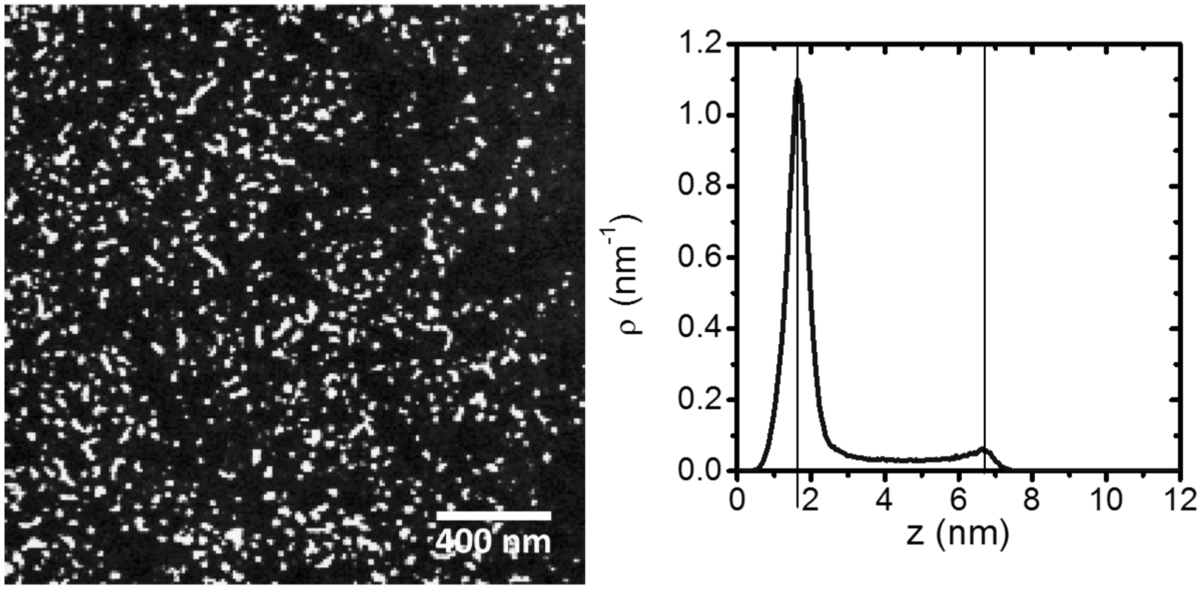

Figure S2: *AFM image of non-metallized DMPG-lipid nanodiscs (height scale 8 nm) and corresponding height distribution function. The vertical lines give the values of the mica (1.6 nm) and the nanodisc height (6.6 nm).*

References

1. Denisov, I. G. Grinkova, Y. V. Lazarides, A. A. & Sligar, S. G. Directed self-assembly of monodisperse phospholipid bilayer Nanodiscs with controlled size, *J. Am. Chem. Soc.* **126,** 3477–3487 (2004).
2. Inagaki, S. *et al.* Modulation of the interaction between neurotensin receptor NTS1 and Gq protein by lipid, *J. Mol. Biol.* **417,** 95–111 (2012).
3. Riske, K. A. Amaral, L. Q. & Lamy-Freund, M. Thermal transitions of DMPG bilayers in aqueous solution: SAXS structural studies, *Biochim. Biophys. Acta - Biomembranes* **1511,** 297–308 (2001).
